# Development, implementation and evaluation of a management specialization course in oncology using blended learning

**DOI:** 10.1186/s12909-020-1957-4

**Published:** 2020-02-06

**Authors:** Raphaella Amanda Maria Leite Fernandes, Jurema Telles de Oliveira Lima, Bruno Hipólito da Silva, Mozart Júlio Tabosa Sales, Flávia Augusta de Orange

**Affiliations:** 1 Comprehensive Healthcare, Instituto de Medicina Integral Prof. Fernando Figueira, Setor de Pós-graduação Stricto Sensu, R. dos Coelhos 300, Boa Vista, Recife, Pernambuco 50070-550 Brazil; 2 Olinda School of Medicine, Recife, Pernambuco Brazil; 3 Oncology Department, Instituto de Medicina Integral Prof. Fernando Figueira, Recife, Pernambuco Brazil; 4 Faculdade Pernambucana de Saúde, Recife, Pernambuco Brazil; 5 Distance Education Center, Instituto de Medicina Integral Prof. Fernando Figueira, Recife, Pernambuco Brazil; 6 Teaching Hospital of the Federal University of Pernambuco, Recife, Pernambuco Brazil

**Keywords:** Continuing education, Professional practice management, Health management, Health education, Distance learning

## Abstract

**Background:**

Identifying effective methods for safeguarding the efficient functioning of the healthcare system contributes significantly towards establishing a successful healthcare organization. Consequently, quality management programs are currently being implemented in healthcare as a vital strategy for patient care. Quality management encompasses protocols and guidelines in decision-making and in the evaluation of processes and treatment flowcharts, data analysis and health indicators, and addresses improvement in the interaction between different health professionals. Qualifying health professionals to perform quality management has represented a barrier to implementing a well-structured management system. Indeed, the pathway to qualifying health managers is often poorly outlined, with clear gaps in the definition of their competencies, training and career plans. Therefore, studies and education-related actions aimed at qualifying health professionals in management are vital if health services of excellence are to be established. The present study aimed to plan, develop, implement and evaluate a management specialization course in oncology using blended learning.

**Methods:**

Following approval by the institution’s internal review board, the study was conducted at the *Instituto de Medicina Integral Prof. Fernando Figueira* (IMIP). The Analysis, Design, Development, Implementation and Evaluation (ADDIE) model was used to plan, develop, implement and evaluate the course. Data were collected as the course participants who had concluded all the modules evaluated the program.

**Results:**

A management course in oncology, consisting of ten sequential modules, was developed and implemented between March 2018 and February 2019. The course consisted of monthly face-to-face encounters, each with 12 h of activities, and distance education using a virtual learning environment. Each module was presented by a specialist on the subject in question. After the end-of-course conclusion work had already been handed in and evaluated by the tutors, the participants completed a form to assess the course using Kirkpatrick’s training evaluation model.

**Conclusions:**

A management course in oncology was developed using the ADDIE model. A high degree of satisfaction was found among the participants regarding improvements in their management skills and their professional behavior. The expectation is that this initiative will ultimately improve healthcare and reduce costs, as well as encourage further innovative educational actions for health professionals.

## Background

In view of its epidemiological, social and economic magnitude, cancer is one of the most complex problems for healthcare systems. Furthermore, the organization of cancer care involves various healthcare levels. Currently considered the second cause of death worldwide, cancer may become the primary cause of death by the year 2030, according to estimates by the World Health Organization [1]. Knowledge on this group of diseases and their treatment has increased enormously in recent years; however, nothing is more far-reaching than health promotion, prevention, early diagnosis and timely treatment [2].

The singularity of care required by cancer patients demands discussing a healthcare model that thoughtfully reflects on how care is provided to these patients. The principal variables that should be the focus of attention when organizing the care of cancer patients include: evidence-based clinical guidelines; a regulated system of access to healthcare; continuity of care; the provision of integrated primary, secondary and tertiary care; comprehensive care; supported self-care; the use of managerial tools for the clinic; management of health conditions and case management; permanent education for health professionals and health education for healthcare users [3–5].

Taking all these variables into consideration, management and continuing education emerge as important tools in the combat of cancer and in the improvement of patient care. Despite major investment in diagnostic and therapeutic technology, the number of cases and cancer mortality rates remain unchanged. In fact, other measures in addition to introducing new treatment modalities could affect the general course of the disease. The health sector is currently in the process of implementing quality management as a vital strategy in patient care [6].

Quality management was initially developed in industry [6–9]. When applied to the health sector, it is intended to direct the focus of the healthcare organization to the needs of the patients, to improving procedures, encouraging team motivation through participation in evaluation and decision-making, and implementing organizational changes based on facts [6].

Nevertheless, the lack of opportunities for qualifying professionals to perform these functions has represented a barrier to the implementation of a well-structured management system. In the field of health, professional training is normally aimed at assuring acquisition of the specific competencies and skills required to exercise the profession, with little focus being placed on learning about quality management [10].

A study conducted in Italy reported that the majority of health professionals were aware of their lack of health management skills and concluded that efforts had to be made to promote more educational interventions in this area [11]. The same investigators showed that education aimed at forming health leaders and managers could reduce disparities and improve health in all social groups, thus achieving greater equity in healthcare [11].

Training in quality management has been shown to result in various benefits with respect to patient care, since the actions implemented are more appropriate to the specific requirements of the health units and are developed by individuals extremely familiar with the organization [12]. However, providing adequate training may also be hindered by individual non-compliance with training tools due to geographical barriers and/or limited available time [13].

The wide use of distance education as a strategy in the continuing education of professionals has successfully increased access to information and democratized education [14]. Indeed, thanks to technological innovations, distance education has been helping individuals already in the job market deal with the disadvantages of distance and time limitations [15].

Consequently, if learning is to be made more available and accessible, new teaching approaches in educational interventions have to be identified and implemented. In this respect, the use of active methodologies is proving a strong learning tool, both at undergraduate and post-graduation level [16].

A specialization course in management within the field of oncology is an important initiative aimed at helping professionals deal with the principal managerial problems involved in administering cancer services and providing better quality and more effective care. The principal objective of this study was to plan, develop and evaluate a management specialization course in oncology through the use of blended learning methodology.

## Methods

This study was conducted at the *Instituto de Medicina Integral Prof. Fernando Figueira* (IMIP), a regional referral unit for cancer treatment. The project was submitted to the institute’s internal review board and approved under reference 2.807.291.

### Study design

This observational cohort study involved an educational intervention consisting of a specialization course in management for health professionals working at oncology clinics within the state of Pernambuco, Brazil. All the professionals were exposed to the educational intervention, which lasted for a total of 10 months. Forty-five days after completion, all participants were evaluated using the first three levels of Kirkpatrick’s four-level training evaluation model to assess their satisfaction, learning and any change in their behavior within the work environment [17].

### Participants and recruitment

Nurses, oncologists, pharmacists, physiotherapists and psychologists, with varying ages and years of experience in oncology, were invited to participate in the course, resulting in a convenience sample of 45 health professionals, all working in oncology clinics in the Brazilian state of Pernambuco. All the participants who finished the course received a certificate of post-graduation.

### Design of the educational intervention

Blended learning, used in the development of the present study, is a strategy nested within the ADDIE model, currently one of the most commonly used and disseminated approaches to instructional design. The ADDIE model was developed by the Florida State University and has been used primarily by the United States armed forces [18, 19]. The acronym ADDIE stands for the five stages of a development process: Analysis, Design, Development, Implementation and Evaluation.

#### Analysis

In the first part of the instructional design process, the context of the course was analyzed and adapted to the needs of IMIP. An extensive review of the literature was performed and the principal points with respect to management in oncology were investigated to ensure that all the fundamental concepts were included in the course. In addition, this content was discussed by a group of six professionals (four physicians, a nurse and an information technology professional) who had conceived the original idea for the course. The literature review and the group discussion thus served to establish the overall aims of the course and the content to be included.

Instruments were developed to evaluate the professionals who participated in the course according to the defined learning objectives, with three cognitive domains being measured: the knowledge, skills and attitude of the participants. Blended learning was partially given in the form of distance learning and partially at face-to-face encounters. After the entire review was complete, the teaching strategies for the face-to-face classes were outlined, based on learning theories, the content to be covered and the learning objectives.

#### Design

**Fig. 1:**
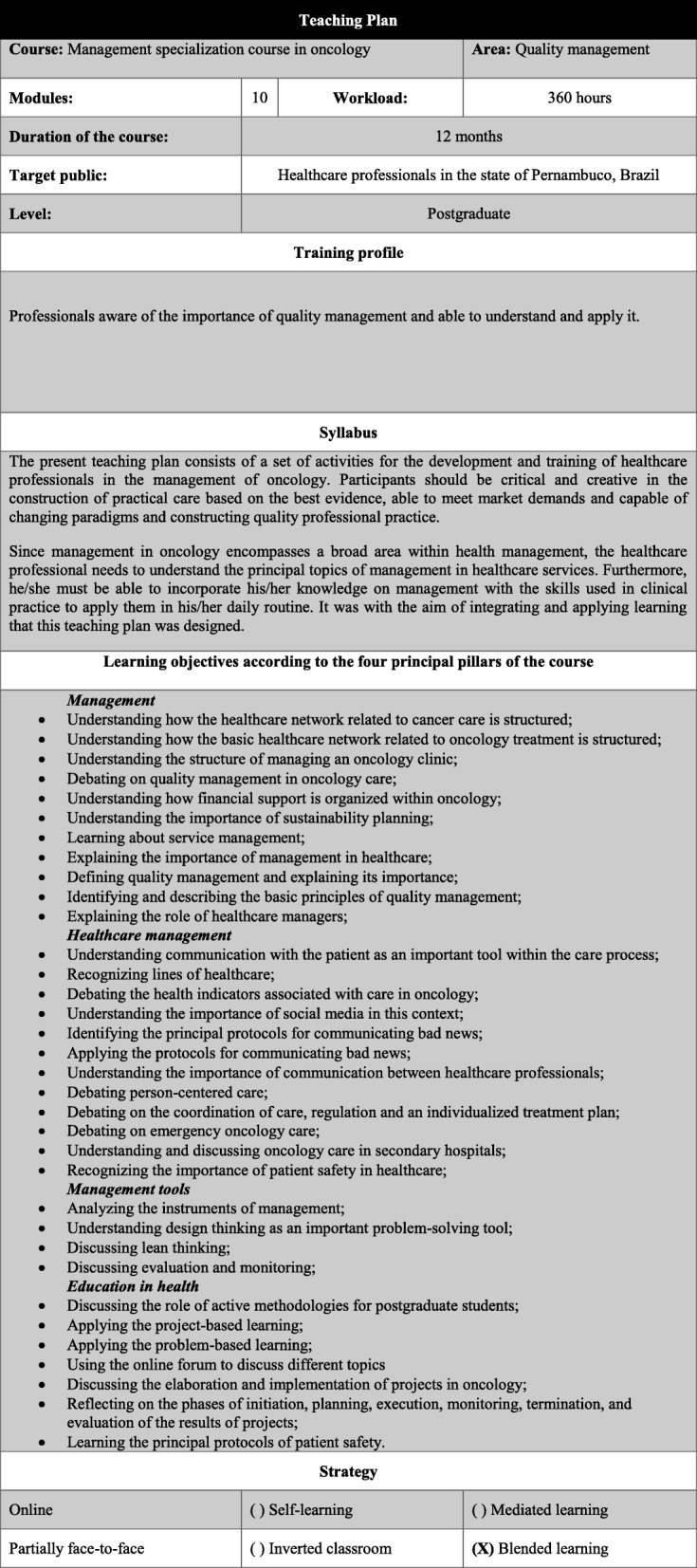
Box showing the contents of the course

In this phase, a course syllabus was designed, including the teaching approach to be used in the course, i.e. the methodology used to allow the participants to achieve the learning objectives and the processes used to evaluate the participants. This material was subjected to a validation process performed by a consensus group consisting of three health professionals, all with a master’s degree in health education. After the course syllabus had been drawn up, the course structure was established, both for the face-to-face encounters and for the distance-learning segment. All participants of the consensus group read and signed an informed consent form. The teaching plan and the contents of the course, including the objectives of learning, the target public, the profile of the participants and the total number of study hours are shown in Fig. 1.

#### Development

In the third stage of the ADDIE, the course was developed using various software programs. Word 2010®, part of the Microsoft Office® package, was used to write the course, while Adobe Captivate® was used to create the teaching materials, with the objective being to make the selected content more dynamic. This software includes a set of functions and resources referred to as rapid e-learning that enables learning objects to be created quicker and more effectively.

#### Implementation

Following development, the course was implemented in modules, the fourth phase of ADDIE. For the application of each module, a professional considered a specialist on the subject of that specific module was invited to participate. To select professionals considered specialists, the validated scale of criteria for the selection of specialists, developed by Guimarães et al. [20], was adapted to the setting of the present study (Table 1). Professionals who achieved at least five points on this validated scale were considered specialists.

**Table 1:** Criteria for selecting specialists, as adapted from the scale proposed by Guimarães et al. [20]

Criteria	Points
At least 4 years’ experience in the specific area	04
At least 1 year’s experience of teaching in the specific area	01
Experience in research with papers published in indexed journals	01
At least 2 years participating in a research group in the specific area	01
PhD in the specific area	02
Master’s degree in the specific area	01
Specialization work in the specific area	01

NB: An extra point was added for each additional year of experience in the area

In addition to applying the modules, the specialists on the subjects in question were able to give their opinions and suggest changes, both regarding the content of the module and with respect to the operational technology platform. All the changes suggested by the specialists regarding the content of the modules were implemented.

#### Evaluation

In the final stage of ADDIE, the participants evaluated the course following its completion. The evaluation was conducted using an online form created by the investigators, based on the literature and validated by two experts in health education. This form contained 27 items categorized into three blocks, aimed at evaluating each one of the first three levels of Kirkpatrick’s evaluation model [17]. The items were rated on a Likert-type scale, with five options of response ranging from “I strongly agree” to “I strongly disagree”.

Kirkpatrick’s evaluation model [17] is widely used to evaluate training programs [21–24]. At the first level, the objective is to evaluate participants’ reactions to the education program in terms of how satisfied they were with the course. The second level is aimed at evaluating how much the participant has managed to learn through the program. The third level evaluates the extent to which behavior and procedures have changed following participation in the course or program. Finally, the last and most complex level investigates the extent to which the program has impacted organizational indicators. This final level was not evaluated in the present study.

The first three levels of the model were applied 2 months after completion of the course to ensure that the entire educational intervention rather than any specific module was evaluated. Furthermore, some time would have had to elapse after conclusion of the course to enable an evaluation to be made regarding whether changes in the participants’ behavior had been incorporated into their routine.

*Evaluation of the first level:* Ten statements related to the student’s motivation, interest and reaction to the course, its professors and methodologies.

*Evaluation of the second level:* Twelve statements related to the learning objectives of the course and how much the student concluded that he/she had learned.

*Evaluation of the third level:* Five statements related to changes in behavior and professional conduct.

To participate in this step, the student had to have read and signed an informed consent form. The inclusion criterion for this step was to have concluded 75% or more of the total number of credit hours for the course. Any participants who had not completed all the items on the evaluation form were excluded from the analysis.

### Data analysis

The questions were scored on a Likert-type scale, with the following options of response: “I strongly agree”, “I agree”, “I neither agree nor disagree”, “I disagree” and “I strongly disagree”. These categorical variables were then transformed into numerical variables. Scores of 1–5 were awarded, with 5 representing “I strongly agree” and 1 “I strongly disagree”. After converting the answers into numbers, the measure of central tendency used was the mode. Results were considered satisfactory for the question when the mode was 4 or 5.

## Results

The final structure of the course was established in 10 once-a-month modules taught face-to-face, each involving 12 h of activities. The classes occurred on a Friday from 6 to 10 pm and on the following Saturday from 8 am-12 pm and from 1 to 5 pm. A syllabus was designed for each one of the ten modules, with the specialists of the subject in question being able to make changes as deemed necessary. The total duration of the course was 10 months. Virtual activities were developed within a virtual learning environment that was part of the course and was available 24 h/day, every day.

### Course presentation

The teaching material used in the course encompassed four principal pillars:

- *Management:* Based on the concepts of management, with a focus on negotiation and decision-making; participatory planning; strategic and system thinking; interpersonal relationships; commitment to results; teamwork and leadership; communication; oncology care policy and strategic planning.
- *Healthcare management:* Based on identifying problems and requirements in management and evaluating healthcare, with a focus on analysis and intervention in the area of health and management of the clinic according to the criteria of efficacy, effectiveness, efficiency, safety and quality in healthcare, line of care, health networks, engagement, interdisciplinarity and the construction and interpretation of health indicators.
- *Management tools:* Introduction to the lean thinking model, a business methodology that seeks to maximize value for customers by eliminating waste. This managerial philosophy is inspired by the practices and results of the Toyota Production System. The aim is to create more value for customers by aligning the necessary steps in the best possible sequence to create value; to perform these steps continuously and increasingly effectively, whenever requested, in the management of the clinic.
- *Education in health:* These aspects were included throughout the course, permitting the development of attributes through the participant’s own experience, involving research methodology, self-management of continuing learning and the use of new technologies.

### Education strategies

The evaluations were designed to include a wide range of cognitive, psychomotor and attitudinal skills. Therefore, the students were evaluated by summative and formative assessment.

All the face-to-face classes involved the use of active methodologies such as problem-based learning, project-based learning, sensory moment, and cultural activities using films and other sensorial materials to reflect on the proposed content. An experienced lecturer proposed ideas on a relevant topic and there were group discussions with a facilitator on a topic covered in a sensory moment or at one of the lectures. In addition, there was a practical activity referred to as a “toolbox” in which the students developed ideas and solved problems using educational and managerial tools. Various formative assessments were made throughout both the face-to-face segment of the course and during distance learning. The students were given feedback on their performance and on the material they were presenting, thus enabling them to review where they had gone wrong and what they had done correctly, and to reconsider their learning processes.

Within the e-learning segment of the course, there were discussion forums with specialists, reading materials that ranged from sites on management in health and education to scientific papers and textbooks. In addition, since one of the methodologies used at the face-to-face encounters was problem-based learning, there was also a space for discussing the problem raised in that module during the e-learning segment in which the students were able to post papers or texts that they had used to back up their learning. In addition, there was an online tutor who acted as a moderator. Another important point of the distance-learning segment was the space for portfolio. In each module, the students reflected on their strongpoints, points that needed improving, their development and learning, the tutors’ performance and the importance of the contents of the module. Based on these factors they put together a reflexive portfolio that was uploaded to the virtual learning environment. This enabled the students to monitor their own reflections, module by module. All these activities involved a total of 24 h per month, making an overall total of 240 h for the whole course.

### Post-training evaluation of the participants using Kirkpatrick’s evaluation model

Forty-eight participants began the course. Of these, 45 (93.75%) completed all the modules and successfully concluded the course and received their certification. All these 45 participants were then invited to complete an online questionnaire. Forty-one of the participants completed the online questionnaire on satisfaction, learning and changes in behavior, the first three levels of Kirkpatrick’s evaluation model. All the questionnaires were completed anonymously.

#### Satisfaction

At this first level of Kirkpatrick’s model, the statements were rated category 4 or 5 in 90% of cases, showing the students’ high rate of satisfaction with the educational intervention (Table 2). For the statement “In my opinion the course was relevant”, all the answers were category 4 or 5, with over 85% being “I strongly agree”, confirming the extent to which the subject of management needs to be elaborated and discussed.

**Table 2:** Likert-type responses for statements on satisfaction: level 1 of Kirkpatrick’s evaluation model

Statement	I strongly agree 5	I agree 4	I neither agree nor disagree 3	I disagree 2	I strongly disagree 1
I enjoyed the course.	68.3%	31.7%	0%	0%	0%
In my opinion the course was relevant.	85.4%	14.6%	0%	0%	0%
I am pleased that I invested my time in training.	75.6%	24.4%	0%	0%	0%
I believe that the course helped me in my professional career.	85.4%	12.2%	2.4%	0%	0%
I participated actively in the course.	56.1%	41.5%	2.4%	0%	0%
The course was motivating.	56.1%	41.5%	2.4%	0%	0%
The professors were well-prepared to apply the modules.	75.6%	22.0%	2.4%	0%	0%
The methodologies used were stimulating.	68.3%	31.7%	0%	0%	0%
The professors motivated me to study.	43.9%	53.7%	2.4%	0%	0%
I pushed myself to learn as much as possible.	68.3%	29.3%	2.4%	0%	0%

#### Learning

In the second level of Kirkpatrick’s model, over 87% of responses were category 4 or 5 for all the statements, showing that the majority of the participants agreed or strongly agreed that they had developed the cognitive, psychomotor and attitudinal skills proposed in the course (Table 3). For statements such as: “I am capable of using design thinking in my routine work” and “I am able to discuss the elaboration and implementation of oncology projects”, responses were in categories 4 or 5 in over 97% of cases, showing that the level of learning reached the higher levels of Bloom’s taxonomy of learning domains [25, 26].

**Table 3:** Likert-type responses for statements on learning: level 2 of Kirkpatrick’s evaluation model

Statement	I strongly agree 5	I agree 4	I neither agree nor disagree 3	I disagree 2	I strongly disagree 1
I know about active methodologies.	36.6%	61%	2.4%	0%	0%
I understand communication with patients as an important tool in the care process.	97.6%	2.4%	0%	0%	0%
I understand about the protocol for giving bad news.	64.3%	29.3%	2.4%	4.9%	0%
I understand how the healthcare network is structured in relation to oncology treatment.	58.5%	39%	0%	2.4%	0%
I understand design thinking as an important tool in problem solving.	78%	22%	0%	0%	0%
I am capable of using design thinking in my routine work.	46.3%	53.7%	0%	0%	0%
I am able to discuss the lean thinking model.	39%	58.5%	2.4%	0%	0%
I am able to discuss the elaboration and implementation of oncology projects.	46.3%	51.2%	2.4%	0%	0%
I understand about quality in oncology care management.	56.1%	43.9%	0%	0%	0%
I recognize the importance of patient safety in healthcare.	95.1%	4.9%	0%	0%	0%
I understand how funding is organized in the area of oncology.	36.6%	51.2%	9.8%	2.4%	0%
I understand the importance of sustainability.	65.9%	29.3%	4.9%	0%	0%
I know about service management.	39%	56.1%	2.4%	2.4%	0%
I am able to discuss evaluation and monitoring.	43.9%	51.2%	2.4%	2.4%	0%

#### Changes in behavior

In the third level of Kirkpatrick’s model, the mode of the responses to all the statements was 4 or 5, showing that a satisfactory change had occurred in the routine and in the behavior of these professionals following the intervention (Table 4). Furthermore, the statement: “I am able to influence my team and teach what I learned in the course” received responses of category 4 or 5 from over 97% of the participants, leading to the conclusion that the educational intervention can extend to more people in addition to the participants.

**Table 4:** Likert-type responses for statements on changes in behavior: level 3 of Kirkpatrick’s evaluation model

Statement	I strongly agree 5	I agree 4	I neither agree nor disagree 3	I disagree 2	I strongly disagree 1
I apply what I learned in the course in my routine work.	51.2%	48.8%	0%	0%	0%
I changed my professional behavior after the course.	58.5%	41.5%	0%	0%	0%
I am aware of a change in my behavior after the course.	61%	39%	0%	0%	0%
I am able to influence my team and teach what I learned in the course.	65.9%	31.7%	2.4%	0%	0%
My colleagues notice that some of my professional attitudes have changed since the course.	26.8%	51.2%	19.5%	0%	2.4%

## Discussion

The present study involved the development of a specialization course aimed at training health professionals in subjects related to management in oncology. The strategies used were active methodology and distance learning, with the objective being to give the student greater autonomy and develop his/her metacognition. The results highlight the effectiveness of this educational intervention within this subject matter.

The field of knowledge chosen for the training of these professionals was carefully selected, since the majority of health-related university courses do not include any modules on health management. This results in professionals who are poorly prepared and insecure with respect to this subject and highlights the need for additional training aimed at developing the necessary skills [27]. Results showed that all the health professionals participating in the present study agreed with the relevance of the proposed subject and were motivated to discuss the topic.

The results following the conclusion of the course in terms of learning, satisfaction and changes in behavior were satisfactory, emphasizing the ease of learning through differentiated teaching approaches involving multimedia-assisted instruction and participatory methods. These findings are in line with the results of other studies on similar education-related subjects [28].

The teaching strategies based on collaborative learning seem to have been important in the present teaching-learning process. Triana et al. stated that collaborative and multidisciplinary strategies should always be used in postgraduate training courses due to the richness of these approaches and the changes that they can make to the behavior of healthcare professionals [29]. The majority of the participants agreed that the learning objectives had been achieved and that the skills had been acquired, and a large percentage of the students reported a change in their professional behavior. These goals are presumed to have been achieved through the teaching approaches used, since the results were very satisfactory with respect to participants’ interest, motivation and satisfaction with the methodologies applied.

In a culture that remains strongly influenced by the robust leadership and management role played by doctors in healthcare teams, it was extremely relevant to see that 97% of the participants, including nurses, physiotherapists, pharmacists and other health professionals, reported a change in their behavior that could influence others in their team. Similar findings were reported from another study conducted in Uganda where an educational intervention was also found to change the behavior of several medical and non-medical health professionals [30].

Although it is impossible to affirm whether any change occurred in hospital management in the institutes in which the course participants worked, it can be said that the training served as a starting point from which to implement organizational changes. This conclusion appears reasonable, considering the positive reactions that highlighted the participants’ self-perception in relation to their acquisition of skills and changes in behavior. A study conducted in the United States reached a similar conclusion regarding the effect of training on patient safety management [31].

Another relevant point that was not, however, evaluated in the present study was the low discontinuation rate among the participants, despite the fact that the duration of the course was considerable, and all the students had a heavy workload to deal with in addition to their studies. Nevertheless, the discontinuation rate was less than 7%. We believe that this fact could reflect the high rate of satisfaction and motivation among the participants, which was a consequence of the use of active methodologies and also of the professionals’ awareness that they needed to work on the subject of management in oncology.

In general, it appears obvious that the intervention was extremely satisfactory in relation to motivation, learning, changes in behavior, compliance and participant approval. In our opinion, these results may be intricately related to the blended learning methodology used, since this is a strategy that results in true gains in the acquisition of skills and knowledge, in addition to reducing the gap between the student and the tutor, permitting greater interaction between the two. This interaction can often increase students’ motivation and reduce the number of students who abandon the course prior to completion [32].

Future studies could evaluate the application of other educational strategies on the subject of management and broaden the scope to health management in general rather than focusing on one single area of health such as oncology, since this is a subject that is so lacking in graduate courses and that exerts a considerable impact on the quality of the services provided.

### Study limitations

The study participants were evaluated up to level 3 of Kirkpatrick’s evaluation model. Level 4, which assesses possible improvements in health indicators and overall results, was not applied. The final level was not evaluated due to time constraints, since the course began in 2018 and concluded in 2019. Ideally, the health indicators should be measured 2 years after the intervention; however, waiting for this additional time would have greatly extended the duration of the study.

## Conclusions

The present study consisted of the development of a specialization course in management in oncology using a blended learning approach aimed at health professionals from the Brazilian state of Pernambuco and using the ADDIE model. The educational intervention proved satisfactory as evaluated using the first three levels of Kirkpatrick’s evaluation model, confirming that blended learning can be used as a teaching approach in continued education at postgraduate level, with excellent levels of client satisfaction. Furthermore, active methodologies can be used in postgraduate teaching, with a high level of student satisfaction.

## Data Availability

All the data generated or analyzed during this study are included in this published article.

## Abbreviations

ADDIE: Analysis, Design, Development, Implementation, and Evaluation model of instructional design.
IMIP: *Instituto de Medicina Integral Prof. Fernando Figueira*
